# Supplementary material for: Biophysical Modeling of Thalamocortical Circuit Dynamics: Species-Specific Insights into Neural Synchrony, Sleep Spindles, and Mechanisms of Neuropsychiatric Disorders
Source: bioRxiv. 2026 Feb 3:2026.02.01.703170. Preprint. [Version 1] doi: 10.64898/2026.02.01.703170 (PMC12889456; doi:10.64898/2026.02.01.703170)
Supplement: Supplement 1 [file NIHPP2026.02.01.703170v1-supplement-1.pdf]

bioRxiv preprint doi: https://doi.org/10.64898/2026.02.01.703170; this version posted February 3, 2026. The copyright holder for this preprint (which was not certified by peer review) is the author/funder, who has granted bioRxiv a license to display the preprint in perpetuity. It is made available under aCC-BY 4.0 International license.

Supplementary Tables:

Current	Neuron Types	Variable(s)	Function
I_{Na+}	TRN, TC, Ctx, IN	m, h	$\alpha_m = \frac{-0.32(V - V_T - 13)}{e^{-\frac{V-V_T-13}{4}} - 1}$<br>$\beta_m = \frac{0.28(V - V_T - 40)}{e^{\frac{V-V_T-40}{5}} - 1}$<br>$\alpha_h = 0.128\, e^{-\frac{V-V_T-17}{18}}$<br>$\beta_h = \frac{4}{1 + e^{\frac{-V-V_T-40}{5}}}$
I_{K+}	TRN, TC, Ctx, IN	n	$\alpha_n = \frac{-0.032(V - V_T - 15)}{e^{-\frac{V-V_T-15}{5}} - 1}$<br>$\beta_n = 0.5\, e^{-\frac{V-V_T-10}{40}}$
I_T	TRN	m, h	$m_\infty(V) = \frac{1}{1 + e^{-\frac{V+52}{7.4}}}$<br>$\tau_m(V) = \frac{0.44 + 0.15}{e^{\frac{V+27}{10}} + e^{-\frac{V+102}{15}}}$<br>$h_\infty(V) = \frac{1}{1 + e^{\frac{V+80}{5}}}$<br>$\tau_h(V) = \frac{22.7 + 0.27}{e^{\frac{V+48}{4}} + e^{-\frac{V+407}{50}}}$
I_T	TC	m, h	$m_\infty(V) = \frac{1}{1 + e^{-\frac{V+65}{7.8}}}$<br>$\tau_m(V) = 0.15\, m_\infty(V)\left(1.7 + e^{-\frac{V+30.8}{13.5}}\right)$<br>$\alpha_1(V) = \frac{e^{-\frac{V+162.3}{17.8}}}{0.26}$<br>$K(V) = \sqrt{0.25 + e^{\frac{V+85.5}{6.3}}} - 0.5$<br>$\tau_2(V) = \frac{62.4}{1 + e^{\frac{V+39.4}{30}}}$<br>$\alpha_2(V) = \frac{1}{\tau_2(V)\,\backslash big(K(V) + 1\backslash big)}$<br>$\alpha_2(V) = \frac{1}{\tau_2(V)\,(K(V) + 1)}$<br>$\frac{dd}{dt} = \alpha_2(V)(K(V)\,(1 - h - d) - d)$

bioRxiv preprint doi: https://doi.org/10.64898/2026.02.01.703170; this version posted February 3, 2026. The copyright holder for this preprint (which was not certified by peer review) is the author/funder, who has granted bioRxiv a license to display the preprint in perpetuity. It is made available under aCC-BY 4.0 International license.

I_H	TC	S_1, F_1<br>S_2, F_2	$H_\infty(V) = \frac{1}{1+e^{\frac{V+68.9}{6.5}}}$ (S_1 & F_1)<br>$\tau_S(V) = e^{\frac{V+183.6}{15.24}}$<br>$\tau_F(V) = \frac{e^{\frac{V+158.6}{11.2}}}{1+e^{\frac{V+75}{5.5}}}$<br>$\alpha_S = \frac{H_\infty(V)}{\tau_S(V)}$<br>$\beta_S = \frac{1-H_\infty(V)}{\tau_S(V)}$<br>$\alpha_F = \frac{H_\infty(V)}{\tau_F(V)}$<br>$\beta_F = \frac{1-H_\infty(V)}{\tau_F(V)}$<br>$\frac{dS_2}{dt} = k_2\,(S_2 - C\,S_1)$

Supplementary Table S1. Voltage-gated and Intrinsic Currents Functions. The gating and time-constant functions are listed for each current, with the corresponding neuron types in which they are expressed: I_{Na+} (fast sodium), I_{K+} (delayed rectifier potassium), I_T (T-type calcium), I_H (hyperpolarization-activated current).

Neuron Type	Currents	$\vec{g}$ mS/cm^2	E_i (mV)	$V_{initial}$ (mV)
Cortex	I_{Na}	50	50	-65
	I_K	5	-90	
	I_L	0.1	-70	
TRN	I_{Na}	100	50	-84
	I_K	10	-90	
	I_L	0.05	-75	
	I_T	1.75	120	
TC	I_{Na}	100	55	-80
	I_K	10	-100	
	I_L	50	-86	
	I_H	0.02	-43	
IN	I_{Na}	100	55	-65
	I_K	10	-100	
	I_L	50	-86	

Supplementary Table S2. Neuron-Type Specific Constants. The maximal conductances ($\bar{g}$), reversal potentials (E_i), and initial voltages ($V_{initial}$) are listed for Cortex (Ctx), thalamic reticular nucleus (TRN), thalamocortical relay nurons (TC), and interneurons (IN).

bioRxiv preprint doi: https://doi.org/10.64898/2026.02.01.703170; this version posted February 3, 2026. The copyright holder for this preprint (which was not certified by peer review) is the author/funder, who has granted bioRxiv a license to display the preprint in perpetuity. It is made available under aCC-BY 4.0 International license.

Current	**α** $M^{-1}S^{-1}$	**β** S^{-1}	**Ei** (mV)
AMPA	3.1842	0.1429	0
$GABA_A$	0.53	0.184	-100

Supplementary Table S3. Ionotropic Synaptic Current Parameter Values. The forward (α) and backward (β) binding rates, and reversal potentials (E_j), are listed for AMPA and $GABA_A$ receptors.

Metabotropic Currents	**K_1** $M^{-1}S^{-1}$	**K_2** S^{-1}	**K_3** S^{-1}	**K_4** S^{-1}	**K_d** μM^2	**E_i** (mV)
MGluR	1.3	6.6	1.9	0.0064	100	0
$GABA_B$	1.3	6.6	0.9	0.0064	100	-100

Supplementary Table S4. Metabotropic Synaptic Current Parameter Values. The rate constants for neurotransmitter binding (K_1) and unbinding (K_2), second-messenger sensitization (K_3) and desensitization (K_4), the dissociation constant (K_d), and the reversal potentials (E_j) are listed for MGluR and $GABA_B$ are provided.
